# Prediction of Higher Heating Values in Bio-Oil from Solvothermal Biomass Conversion and Bio-Oil Upgrading Given Discontinuous Experimental Conditions

**DOI:** 10.1021/acsomega.3c04275

**Published:** 2023-10-04

**Authors:** Abraham Castro Garcia, Phoebe Lim Ching, Richard HY So, Shuo Cheng, Sasipa Boonyubol, Jeffrey S. Cross

**Affiliations:** † Department of Transdisciplinary Science and Engineering, School of Environment and Society, Tokyo Institute of Technology, 2-12-1 S6-10, Ookayama, Meguro-ku, Tokyo 152-8552, Japan; ‡ Bioengineering Graduate Program, Chemical and Biological Engineering Department, Hong Kong University of Science and Technology, 999077, Hong Kong; § Department of Industrial Engineering and Decision Analytics, Hong Kong University of Science and Technology, 999077, Hong Kong

**Keywords:** bio-oil, biomass, catalyst, machine learning, higher heating value

## Abstract

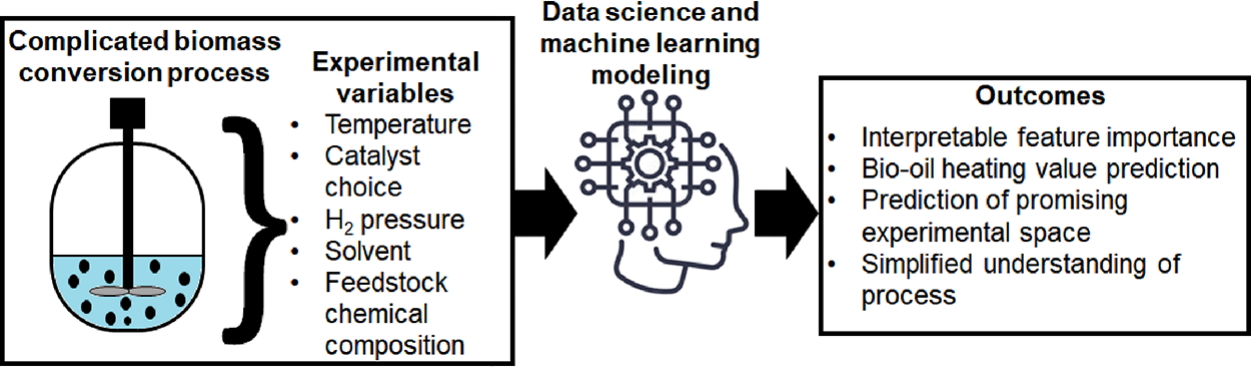

Both the conversion of lignocellulosic biomass to bio-oil (BO) and the upgrading of BO have been the targets of many studies. Due to the large diversity and discontinuity seen in terms of reaction conditions, catalysts, solvents, and feedstock properties that have been used, a comparison across different publications is difficult. In this study, machine learning modeling is used for the prediction of final higher heating value (HHV) and ΔHHV for the conversion of lignocellulosic feedstocks to BO, and BO upgrading. The models achieved coefficient of determination (*R*^2^) scores ranging from 0.77 to 0.86, and the SHapley Additive exPlanations (SHAP) values were used to obtain model explainability, revealing that only a few experimental parameters are largely responsible for the outcome of the experiments. In particular, process temperature and reaction time were overwhelmingly responsible for the majority of the predictions, for both final HHV and ΔHHV. Elemental composition of the starting feedstock or BO dictated the upper possible HHV value obtained after the experiment, which is in line with what is known from previous methodologies for calculating HHV for fuels. Solvent used, initial moisture concentration in BO, and catalyst active phase showed low predicting power, within the context of the data set used. The results of this study highlight experimental conditions and variables that could be candidates for the creation of minimum reporting guidelines for future studies in such a way that machine learning can be fully harnessed.

## Introduction

It is foreseen that liquid hydrocarbons will still be part of our lives for the foreseeable future, due to several key technologies that cannot be electrified or powered by alternative sources, such as fuel for aviation, heavy trucks, and maritime vessels.^1^

To overcome this, extensive research has been devoted to the conversion of renewable biomass resources that can potentially be transformed into molecules that can fulfill the role that fossil fuels currently serve.^2^ Great success has been found in the conversion of edible biomasses such as vegetable oils and simple carbohydrates to biodiesel^3^ and ethanol,^4^ respectively. However, these have attracted criticism for their potential impact on food prices, and thus, the conversion of nonedible biomass has been promoted.^5^

Among nonedible biomass, lignocellulosic biomass is the most abundant. However, due to its recalcitrance, high-temperature thermochemical conversion methods are used to convert it to more useful forms, such as bio-oil (BO) or syngas.^6^ The production of BO from lignocellulosic feedstocks has seen large progress especially through solvothermal conversion methods that allow the use of moderate temperatures and often results in BO with a better higher heating value (HHV), which is key for fuel purposes.

**Figure 1:**
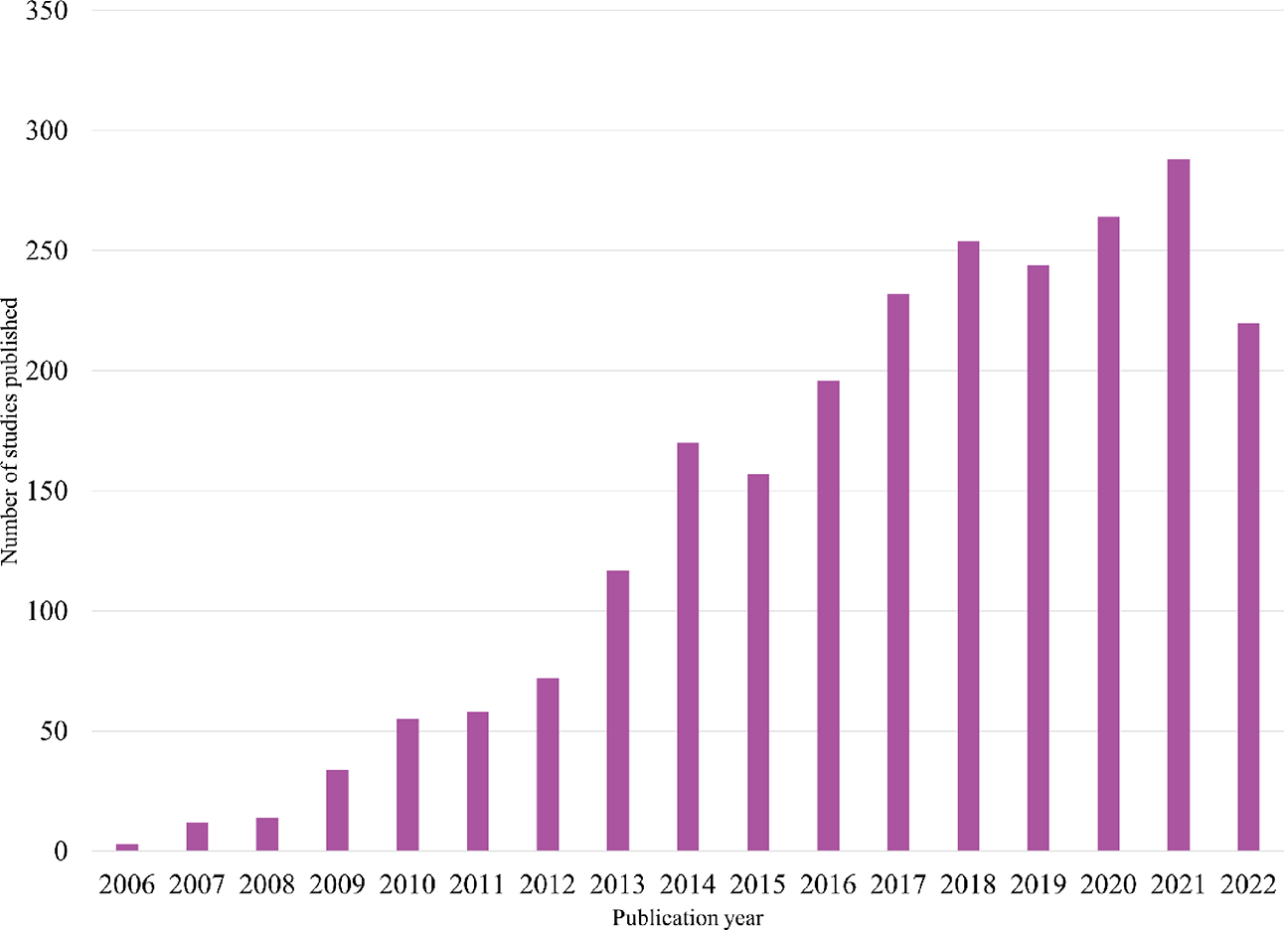
Publication trend of studies related to bio-oil upgrading found in Web of Science using bio-oil upgrading as the search string.

This BO, nevertheless, still requires to be upgraded to improve its HHV and other fuel properties such as viscosity and corrosiveness, by reducing its oxygen content by using organic solvents and hydrogen gas, usually in the presence of a catalyst.^7^ The reactions associated with the upgrading of BO fall under the umbrella term of hydrodeoxygenation (HDO), wherein oxygen is removed by the action of hydrogen through hydrogenation or hydrogenolysis.^8^ However, compared to the hydrocarbon mixtures found in crude oil, the biomass and BO contain a higher diversity of molecules and structures and it is not easy to keep track of which reactions are happening. The distribution of chemicals found in the BO is dependent on the properties of the lignocellulosic biomass feedstock used to produce it, with lignin-heavy feedstocks resulting in higher concentrations of aromatic chemicals and cellulose being converted into ketones, furans, and sugars.^9^ This variation in lignocellulosic biomass composition in addition to the great diversity of experimental choices such as solvents, catalysts, the presence or absence of hydrogen gas, and reaction conditions results in a large number of possible experiments. This also resulted in extensive research that has become more popular in recent decades, as seen in Figure 1, where the number of results for the search string bio-oil upgrading in Web of Science shows a sudden increase at the start of 2010s.

Many studies on the topic of “biomass to bio-oil” and “bio-oil upgrading” have very different methodologies that are often not necessarily justified in terms of why they choose certain reactants, process conditions, or strategies in their experiments.

Typical types of studies in solvothermal biomass conversion to BO or BO upgrading include:

- Noncatalyzed solvolysis/upgrading in water or mixtures of organic solvents and water^10^
- Solvolysis catalyzed by metallic heterogeneous catalysts in the presence of H_2_ gas
- Processing with nonstandard heating methods, such as microwaves^11^
- BO or solid feedstock (SF) being either untreated woody biomass^12^ or isolated lignin^13^
- Usage of low (∼200 °C)^8^ or high temperatures (350 °C−)^14^

In turn, this makes the studies hard to compare and results in slow progress in this research field, as extrapolations from very different studies do not seem superficially compatible.

Of the metrics evaluated for the production of BO and its upgrading, HHV is often pointed as the most important, with viscosity, corrosiveness, and other fuel properties largely correlating in a positive way with the increase of HHV.^15^ In addition to this, a higher HHV BO is more economically valuable as it can be used on higher-standard combustion engines of different kinds.

Recent advances in the usage of machine learning have allowed for the development of models that can predict HHV in raw biomass^16^ using data from proximate analysis, torrefied biomass as a function of the treatment conditions,^17^ and BO derived from hydrothermal liquefaction of wet biomass and wastes,^18^ providing not only good prediction performance, with *R*^2^ scores ranging from 0.83 to 0.93, in spite of relying on small, human-curated data sets originating from literature. In addition to the prediction performance, interpretation of the models made was also possible through the use of partial dependency plots^18^ or SHAP values,^17^ obtaining insight into how the features in the model impact the phenomena or properties responsible for the values obtained.

Inspired by these studies, in this work, knowledge of chemical engineering and machine learning was combined. Specifically, we used knowledge on the biomass liquefaction processes to construct a data set based on studies that share common experimental conditions and variables. Then, machine learning was used to bridge the differences in feedstock, solvent choice, catalyst active media, and experimental variables, which cannot be accomplished by simple linear or curve fitting. The resulting models can predict the change in HHV in biomass-to-bio-oil processes and the upgrading of the BO by relying on data extracted from the literature, obtaining simultaneously variable importance that provides insight into the mechanisms involved in the processes as well as useful observations for future research on BO production and upgrading. The results in this study demonstrate that a few processing conditions across studies have the biggest impact on the resulting HHV of the BO produced or upgraded. To date, this is the first study to predict the increase in HHV of BO as a function of its upgrading process conditions.

## Materials and Methods

### Data Collection and Preprocessing

Many process variables are known to affect biomass-to-bio-oil and BO upgrading. Based on the extensive consultation on the literature and previous work,^19^ the studies that report the experimental data outlined in Table 1 were gathered, including HHV which was used as the target of the predictive model. These features were chosen based on comprehensive analysis of the different measurable process parameters described across studies on this topic. The change in HHV from the original biomass to BO, as well as the change in BO before and after upgrading, was denoted as “ΔHHV”. After this, an exhaustive search of the literature was carried out to look for studies that focused on solvothermal upgrading of biomass to BO and BO upgrading. This was done by using the following search strings in Web of Science in July of 2022:

- Solvolysis biomass
- Bio-oil upgrading

**Table 1:** Machine Learning Features and Label Names along with Their Descriptions

**feature and label names**	**description**
Elemental composition (wt %)	Concentration of C, H, O, and N elements in the feedstock
BO/SF original HHV (MJ/kg)	Original higher heating value of the feedstock, measured or calculated from the elemental composition
aCatalyst name	Active phase of the catalyst used in the experiment
aSolvent name	Solvent name
Solid feedstock name	Feedstock name, all wood and grass varieties were grouped together
Reaction time (min)	Reaction time in minutes
Temperature (°C)	Temperature in °C
Active metal/solvent ratio (mg/mL)	Ratio of active metal in catalyst to solvent
Active metal/feedstock ratio (mg/mg)	Ratio of active metal in catalyst to feedstock
Catalyst metal/solvent ratio (mg/mL)	Ratio of catalyst to solvent
Catalyst metal/feedstock ratio (mg/mg)	Ratio of catalyst to feedstock
Feedstock/solvent ratio (mg/mL)	Ratio of feedstock to solvent
H^+^ ion added (mol)	Moles of H^+^ added as strong acid equivalent
H_2_ pressure factor (MPa H_2_·mL)	Estimation of H_2_ gas used, defined as the product of the pressure of H_2_ and the “difference between reactor volume and solvent volume”
Reactor volume – solvent volume (mL)	Defined as the reactor volume minus the volume of solvent used
bFinal HHV (MJ/kg)	Final HHV value of the BO, measured or calculated
bΔHHV (MJ/kg)	Change of HHV value of the BO, measured or calculated

a This variable was one-hot encoded due to its categorical nature.

b Final HHV and Δ*H*HV were the labels in this study.

This resulted in initial numbers of 172 and 2,392 documents, respectively, that were then screened to make sure they reported as many of the process variables noted in Table 1. After the screening process, a total of 15 and 29 papers were selected that fit the criteria, resulting in a total of 175 and 211 data points, respectively.^20−61^ Studies that were not selected were deemed unfit due to noncompatible methodologies or underreporting of results and process conditions. This search for data and processing centers exclusively on changes to HHV or resulting HHV in BO from experiments but not directly on the chemical species found in the BO, which is beyond the scope of this work. All methodologies from selected papers were carefully analyzed for compatibility of results among each other.

**Figure 2:**
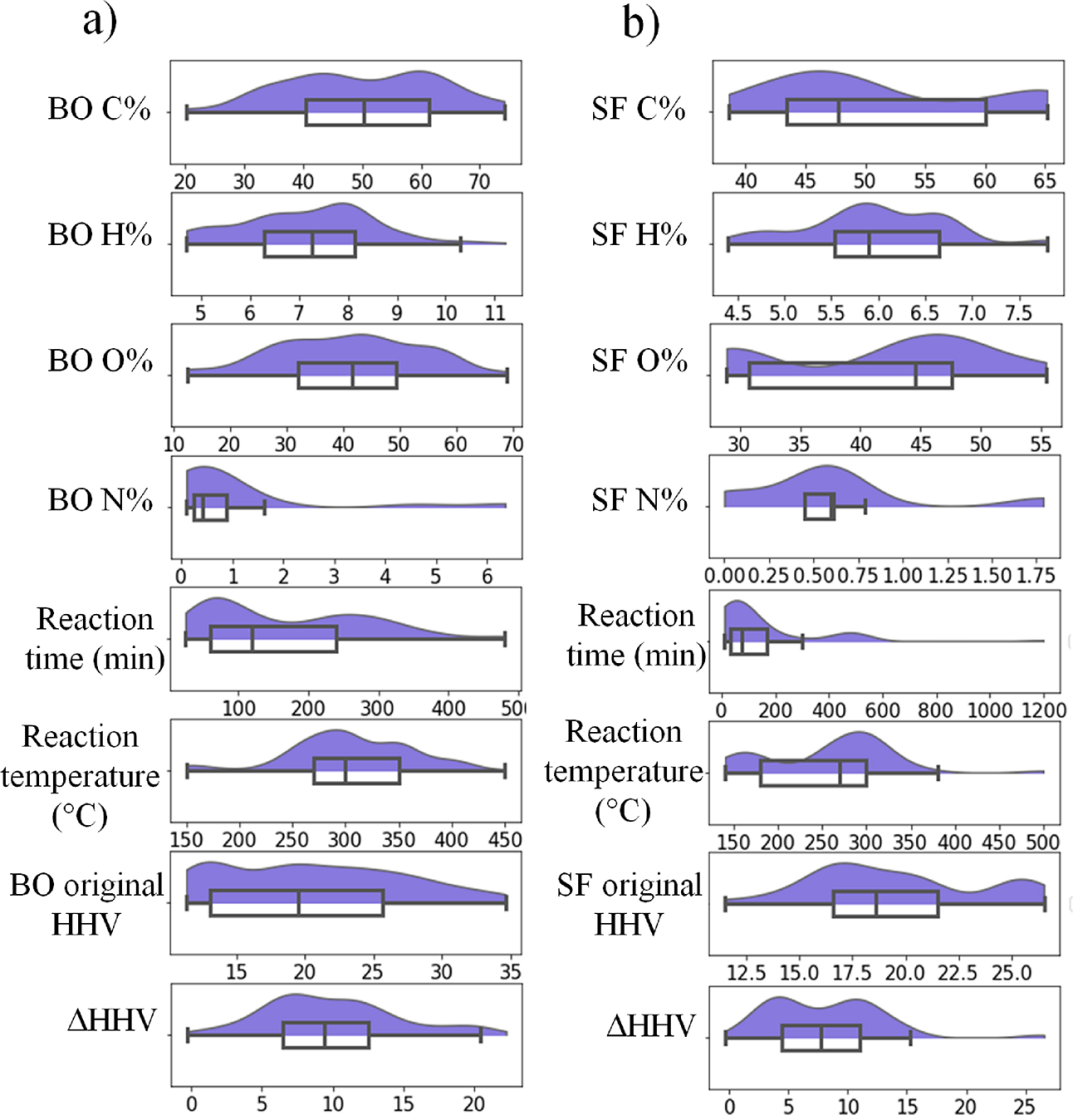
Violin distribution and box-plots for elemental composition, reaction time, reaction temperature, original HHV, and change in HHV after processing for (a) BO and (b) SF.

From the data captured, the distribution of values for elemental concentration, reaction time, reaction temperature, original HHV value, and change in HHV value after processing is shown as violin and box-plots in Figure 2a for BO and Figure 2b for SF. These features were represented as violin plots due to their direct impact in the outcome of a given experiment.

Here, it can be observed that distributions of the features and labels are most often not normally distributed. Although most machine learning methods do not rely on assumptions of data normality to work properly, it is clear that the measures of correlation such as Pearson’s correlation matrix would not work properly (because it relies on data normality).

### Machine Learning Method Used and Evaluation Indicators

The processes in this study were modeled by using an Extreme Gradient Boosting (XGBoost) machine. It must be noted that other ML methods were also tested; however, XGBoost showed marginally higher performance. XGBoost has been extensively applied in modeling-related processes. This work opts to highlight two main attributes of XGBoost as a modeling approach, which makes it a strong candidate for the intended application. First, XGBoost is an ensemble of regression trees (visually summarized in Figure 1). Being part of the family of ensemble models means that the XGBoost model is typically composed of hundreds of regression trees, which each make a partial estimate of the variable being predicted, i.e., ΔHHV or final HHV in this case.

**Figure 3:**
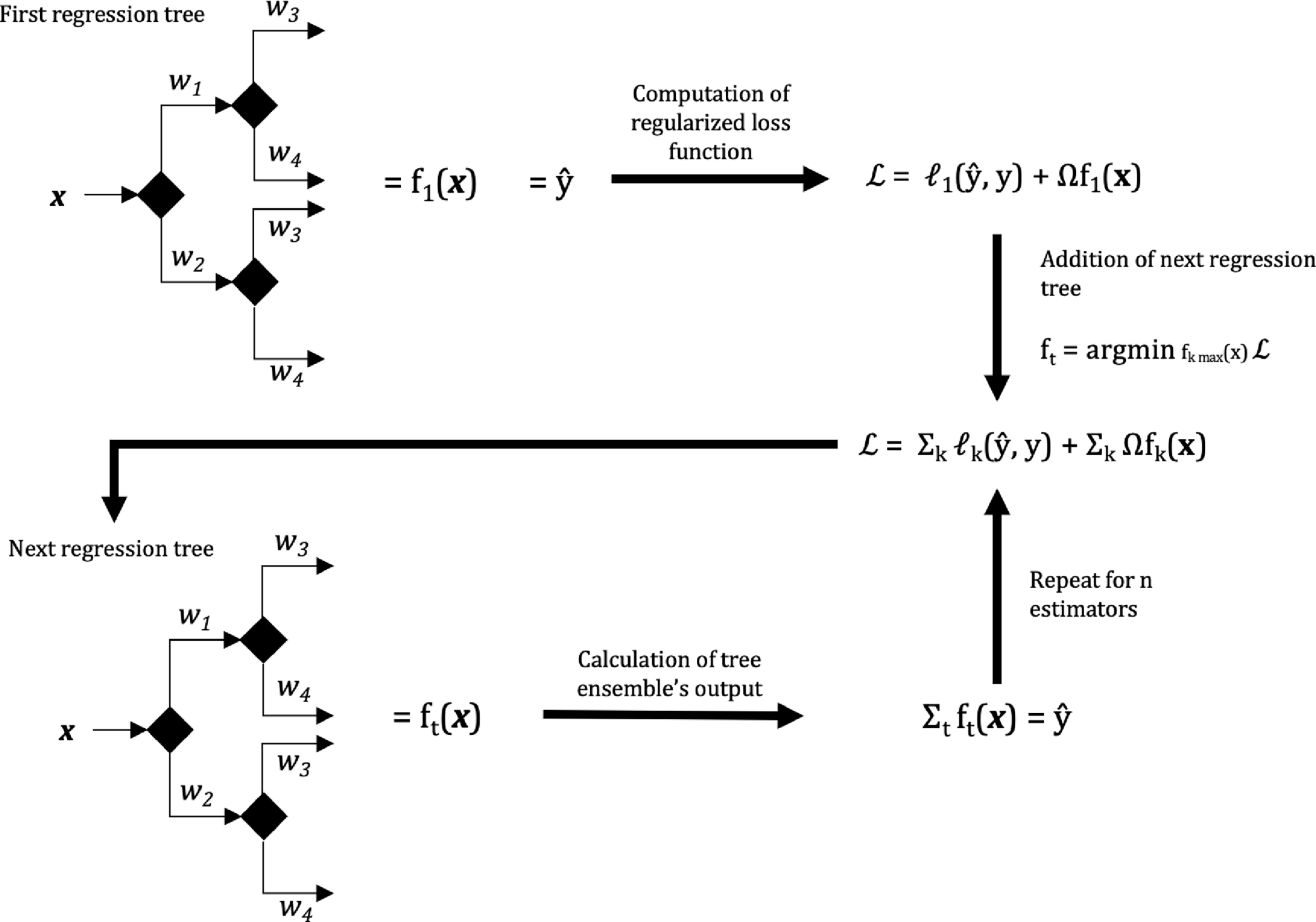
Visual representation of the learning process of XGBoost.

Regression trees are composed of layered “branches” and scored “leaves” (leaf weight, *wk*), as shown in Figure 3. At each branch, a data point is assigned to a leaf or branch in the next layer, according to the value of a certain feature. Features that are relatively important to the predicted variable will assume this role in many branches. The last layer of branches is assigned to a leaf, with the continuous score assigned to this leaf serving as the contribution of that regression tree to the predicted variable. Individually, the regression trees are “weak learners”, being oversimplified models and having a tendency for overfitting because of their structure. However, when the output of these models is aggregated, gross errors and noise can be averaged out while consistent inferences across many regression trees are highlighted.

In particular, a regression tree that assigns conditional scores in the manner described herein is well-suited to the data in this study. This study uses potentially heterogeneous data collected from multiple studies that may have implicit and explicit differences. The examples of implicit differences include undocumented details about the experimental methodology (e.g., purity of reactants used, number of effective catalytic active sites in the catalyst, or differences in workup during experiments), while the examples of explicit differences include differences in feedstock and catalyst, which are clearly identifiable. Most likely, these differences would result in a skewed, multimodal, or otherwise, non-normal distribution, which makes them unsuitable for most of the models (e.g., fitted lines or curves). Regression trees are not under any assumption of a probability distribution and are mostly deemed appropriate for these applications.

The second reason why XGBoost was selected for this study is that its assignment of scores acknowledges the potential of sparse data sets. In general, sparse data sets are those with many 0 elements. This is a common phenomenon in machine learning. In the context of application in this research, some data points may be missing one or more features, as the documented factors and parameters differ from study to study. In addition, categorical variables such as the solvent type or the catalyst type need to be one-hot encoded, which means an integer 0 and 1 is assigned to indicate if a data point uses a particular solvent. The presence of 0 values in the data set tends to be a problem for most models, which must consider the zeroes as a continuous variable. In that sense, the high frequency of zeros could make the mean magnitudes of certain parameters seem lower. Parametric methods that are reliant on these statistics would thus be skewed in response to the presence of the 0s. On the other hand, XGBoost is sparse-aware in the sense that, in its assignment of a next branch or leaf, a specific assignment can be made for the 0 value, thus allowing it to accommodate missing and one-hot encoded data. Following the addition of the final regression tree, the predicted value *ŷ*_*i*_ can be represented as the sum of the weighted regression trees (*f*_*t*_(***x***)) as in eq 1.
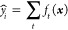
1

The performance of the model was evaluated by using the coefficient of determination (*R*^2^) and root mean square error (RMSE), whose eqs 2 and 3 are shown below, respectively.
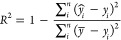
2
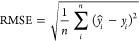
3where *n* represents the number of test samples, *Y*_*i*_^exp^ denotes the experimental value, and *Y*_*i*_ represents the predicted value. *Y*_avg_^exp^ represents the mean value of *Y*_*i*_^exp^ and *Y*_*i*_, respectively.

Data for all models was split for 80% training and 20% testing, and all models used *n*_estimators = 5000, with all other hyperparameters being set to were taken from defaults in the Scikit-learn libraries. *K*-fold validation was used to evaluate the model performance.

### Feature Importance Calculation

Feature importance for the models was obtained by using the SHapley Additive exPlanations (SHAP) values.^62^ Each feature has a corresponding calculated SHAP value representing the contribution of that feature toward the prediction. These values are based on the marginal contribution of the feature or the difference between the values predicted by a model including that feature and one without it. The difference may be either positive or negative, indicating whether that feature makes a positive or negative contribution to the prediction. A multivariate model such as this research can be decomposed into many models by using different combinations of features as inputs. As such, SHAP values are the weighted sum of the marginal contributions of each feature to the prediction. This is illustrated by eqs 4, 5, and 6.
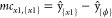
4
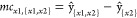
5
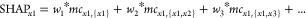
6
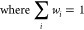


Each data point has a corresponding predicted value that corresponds to its SHAP value. The SHAP values for the data set can be interpreted collectively to understand the general behavior of the model for different inputs. This can be used to confirm the logic of the model, i.e., whether it follows the known or hypothesized effect of certain parameters on HHV. It can also confirm that less important features have zero contribution instead of adding noise to the prediction. As the calculated SHAP values follow a continuous scale, the SHAP values of one-hot-encoded categorical features cannot be reliably interpreted and were excluded from the analysis.

## Results and Discussion

### Evaluating Prediction Accuracy

This study consists of four models, given two predicted values and two processes (biomass solvolysis to produce BO and BO upgrading). The accuracy of each model was evaluated based on common error measures (i.e., *R*^2^, RMSE) and the linear plot of predicted and reported values. The error measures indicate good fitting of the XGBoost model for final HHV and ΔHHV prediction in both the training and test sets. The *R*^2^ values range from 0.96 to 0.99 (training) and 0.77–0.86 (test) across the four models. The RMSE, which corresponds to the average difference in real units of HHV, ranges from 0.42 to 0.89 MJ/kg (training) and 1.78–2.16 MJ/kg (test). A summary of the error measures is given in Table 2.

**Table 2:** Accuracy/Error Measures for Final HHV and ΔHHV Prediction

	**Training**		**Test**	
	*R*^2^	RMSE (MJ/kg)	*R*^2^	RMSE (MJ/kg)
**Solvolysis of lignocellulosic SF**				
Final HHV prediction	0.99	0.42	0.83	1.94
ΔHHV prediction	0.99	0.42	0.79	1.78
**BO upgrading**				
Final HHV prediction	0.97	0.89	0.86	2.12
ΔHHV prediction	0.96	0.89	0.77	2.16

**Figure 4:**
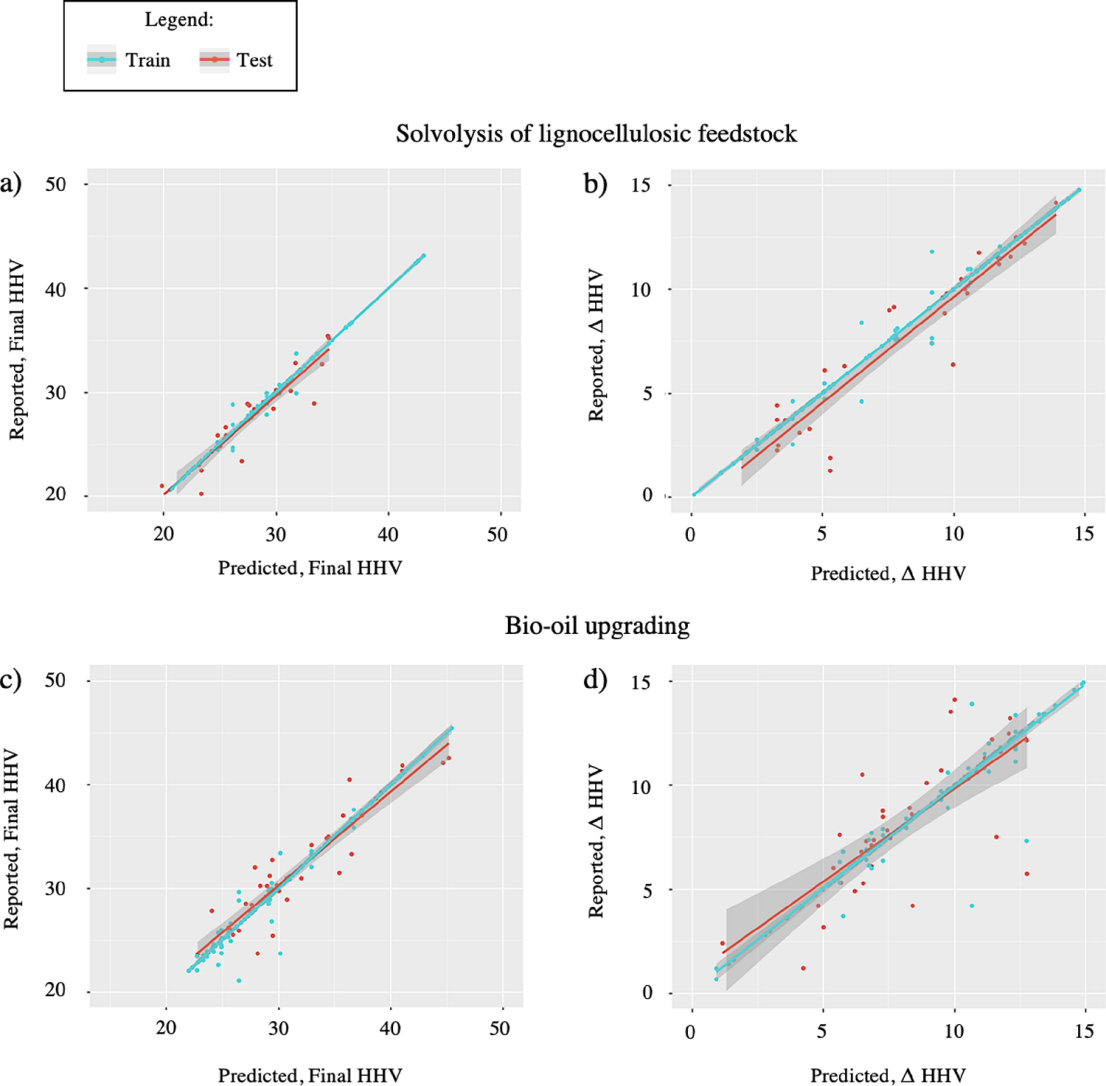
Model performance for the final HHV and ΔHHV for lignocellulosic SF conversion to BO through solvolysis and BO upgrading. (a) Final HHV for solvolysis BO. (b) ΔHHV for solvolysis BO. (c) Final HHV for BO upgrading. (d) ΔHHV for BO upgrading.

In Figure 4, the linear plots of the predicted and reported HHV values are used to provide more insight into the sources of error. For solvolysis, there are a few predicted values with a large deviation from the real reported value, which skews the entire RMSE. Specifically, some data points for the final HHV are significantly overpredicted on the lower end of the regression line, indicating insufficient data for the lowest HHV values. Conversely, using ΔHHV shows a wider variance in error values yet a lower error magnitude as a whole. As the final HHV can be derived from ΔHHV and the initial HHV, the latter model may be more reliable for making estimations on HHV. On the other hand, the regression lines for BO upgrading indicate a tendency to underpredict lower values and overpredict higher values. This is more evident for the ΔHHV, while all data points for the final HHV adhere to the regression line except for a few outliers.

### Evaluating the Model’s Logic and Interpretability

**Figure 5:**
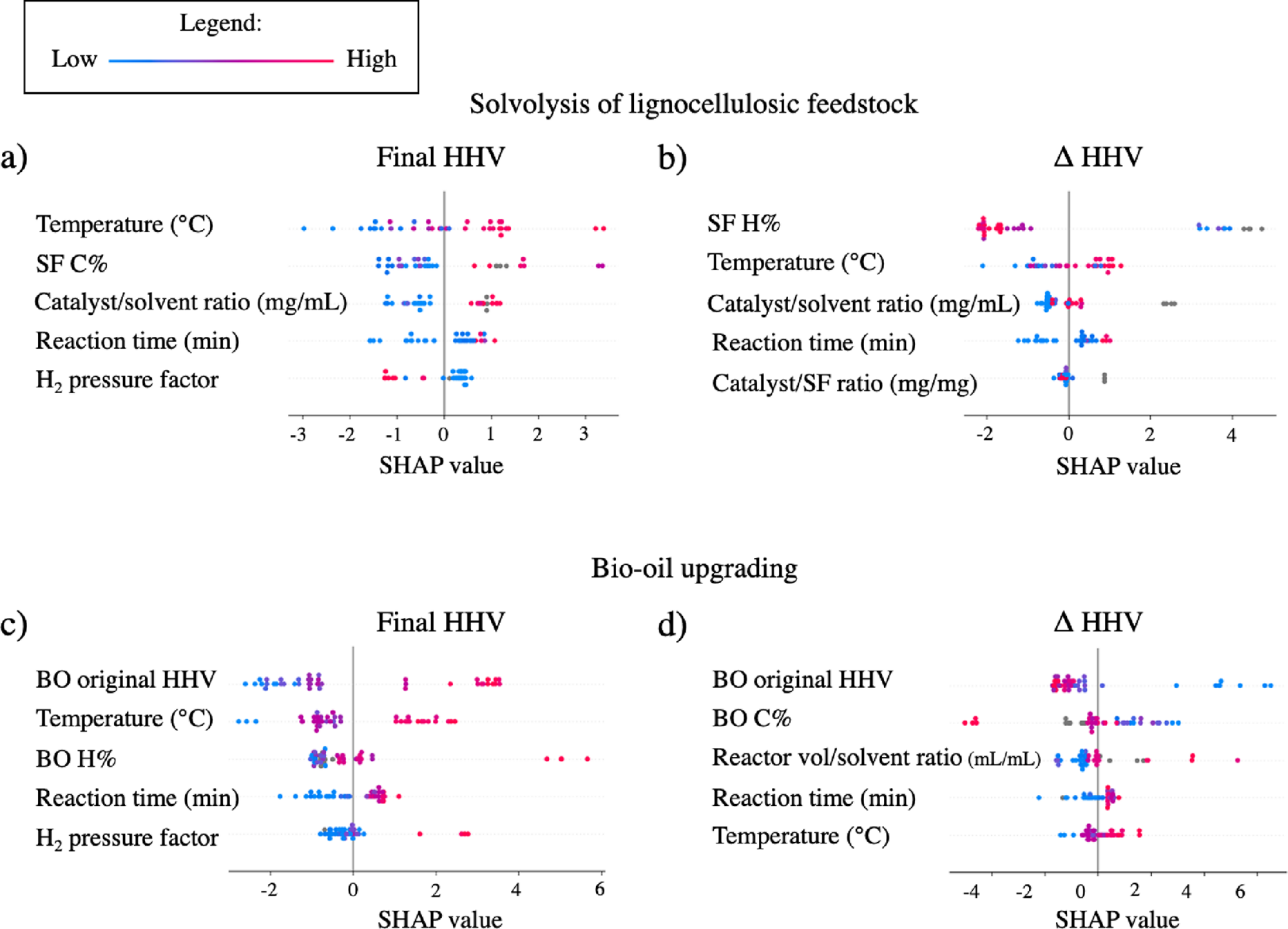
SHAP values for (a) final HHV and (b) ΔHHV from solvolysis of lignocellulosic SF. SHAP values for (c) final HHV and (d) ΔHHV from BO upgrading.

SHAP values were used to understand the model’s logic for prediction. In Figure 5, the beeswarm plots of the most important variables for the models are displayed for both final HHV and ΔHHV of solvothermal conversion to BO of lignocellulosic SF to BO in (a–d) for BO upgrading.

In Figure 5a, we can observe a clear tendency for final HHV. The value of the final HHV increases with the increasing temperature, and the decrease in temperature is associated with the lower final HHV in the produced BO. In the context of the data used to train the model, this could be explained by the reactions associated with the removal of oxygen, such as hydrogenolysis and hydrogenation, which are more prevalent in the temperature range of 250–350 °C.^63^

The concentration of elemental carbon in the original biomass is shown to be important for predicting the final value of HHV of the produced BO. Its importance is largely a coconsequence of the zero-sum relationship in elemental composition, meaning that higher carbon content is associated with higher hydrogen content and lower oxygen content. It is important to note that a high starting oxygen content in the feedstock does not necessarily mean that the final HHV of the resulting BO will be low but rather that the conditions of the solvothermal process would have to be tailored to remove more oxygen. Longer reaction times are associated with higher final HHV of the BO, which stands to reason, as if oxygen-removing reactions continue to take place for a longer time, that it should result in lower final oxygen content and, therefore, higher final HHV. However, at the right reaction conditions, short reaction times can still result in a high final HHV of the resulting BO, depending on the combination of reactants and process conditions. The extended reaction time may be detrimental to the yield of BO in cases where lignin-derived compounds can repolymerize into higher molecular weight fragments that form char.^64^ This can be prevented by the usage of short-chain alcohols such as methanol and ethanol as solvents in the reaction, due to their ability to react with lignin-derived reaction intermediaries and prevent the formation of char,^65^ examples of which represent a large part of the used data set.

The role of catalyst to solvent ratio can be an issue of reaction optimization depending on the choice of heterogeneous catalyst or the absence of it. Many of the transition metal species found in the experiments that compose the data set used can react with the short-chain alcohols used (methanol or ethanol) to generate hydrogen^66^ or promote alkylation reactions^67^ that ultimately result in higher final HHV of the BO produced. It must be pointed out that there are a significant number of noncatalyzed experiments in the data set used, in which case the ratio of catalyst to solvent was defined as 0; thus, the above observations would not apply in these cases. The distribution of the H_2_ pressure factor indicates that the lower the number (less moles of hydrogen), the higher the resulting final HHV should be. This goes against the common understanding that more H_2_ gas should result in higher HHV, due to the removal of oxygen in the BO. This unexpected pattern may be due to the sparse distribution of values for this variable found in our data set, which can be observed in the violin plot for the H_2_ factor found in the supplemental file.

Figure 5b shares many parallels with Figure 5a such as elemental starting elemental composition, reaction time, and temperature. However, the interpretation of the catalyst/solvent ratio is more difficult in this case, as the values are closely clustered. The ratio of catalyst/feedstock shows an ambiguous trend, with both low and high values sometimes being associated with lower ΔHHV. This is probably due to the presence of multiple optimal ratios of catalyst/feedstock in the data set from different studies.

Figure 5c shows that the starting HHV of the BO holds the highest importance in the prediction of the final HHV. High starting HHV in BO is strongly associated with low final HHV from the upgrading process. This is due to the strong correlation between the oxygen content and HHV, where high starting HHV values go hand in hand with low oxygen content. In a similar vein, the carbon content in the BO is a strong predictor for the final HHV of the BO, due to the relation between elemental composition and HHV. Regarding the temperature, a clear correlation between higher temperature and a higher resulting final HHV can be observed. The temperatures in the high quartiles are positively associated with higher resulting final HHV, which falls in the temperature range of 250–350 °C previously mentioned. The distribution of the H_2_ pressure factor also shows a clear relation to the final HHV of the BO, where low values (a smaller number of moles of hydrogen used) result in low final HHV. With regard to the reaction time, it is clear that longer reaction times are associated with higher final HHV.

For Figure 5d, the BO original HHV plays an important role in the resulting ΔHHV. This can be observed from the large cluster of SHAP values on the left side of Figure 5d. From the perspective of the chemical components of the BO, it makes sense that if a given BO sample already contains little remaining oxygen, the resulting possible increase in HHV depends on how much oxygen remains to be removed. This can also be seen that the high carbon content in the original BO was also strongly associated with lower final HHV. Temperature, on the other hand, displays a different spread of SHAP values, which can be seen in the final HHV case for BO upgrading (shown in Figure 5c), still ultimately following a similar trend where higher temperature results in higher ΔHHV. The relation between the ratio of solvent volume to reactor volume was intended to be used as an approximation of the process pressure, on the assumption that the resulting pressure at high temperature is mostly a consequence of the solvent at high temperature and not because of gas being generated. Based on this assessment, it appears that lower process pressure can be associated with higher ΔHHV. However, a significant number of points also cluster around the SHAP value of 0, indicating that there are circumstances where the feature has no impact. Last, the reaction time displays a very similar pattern as shown in Figure 5c).

**Figure 6:**
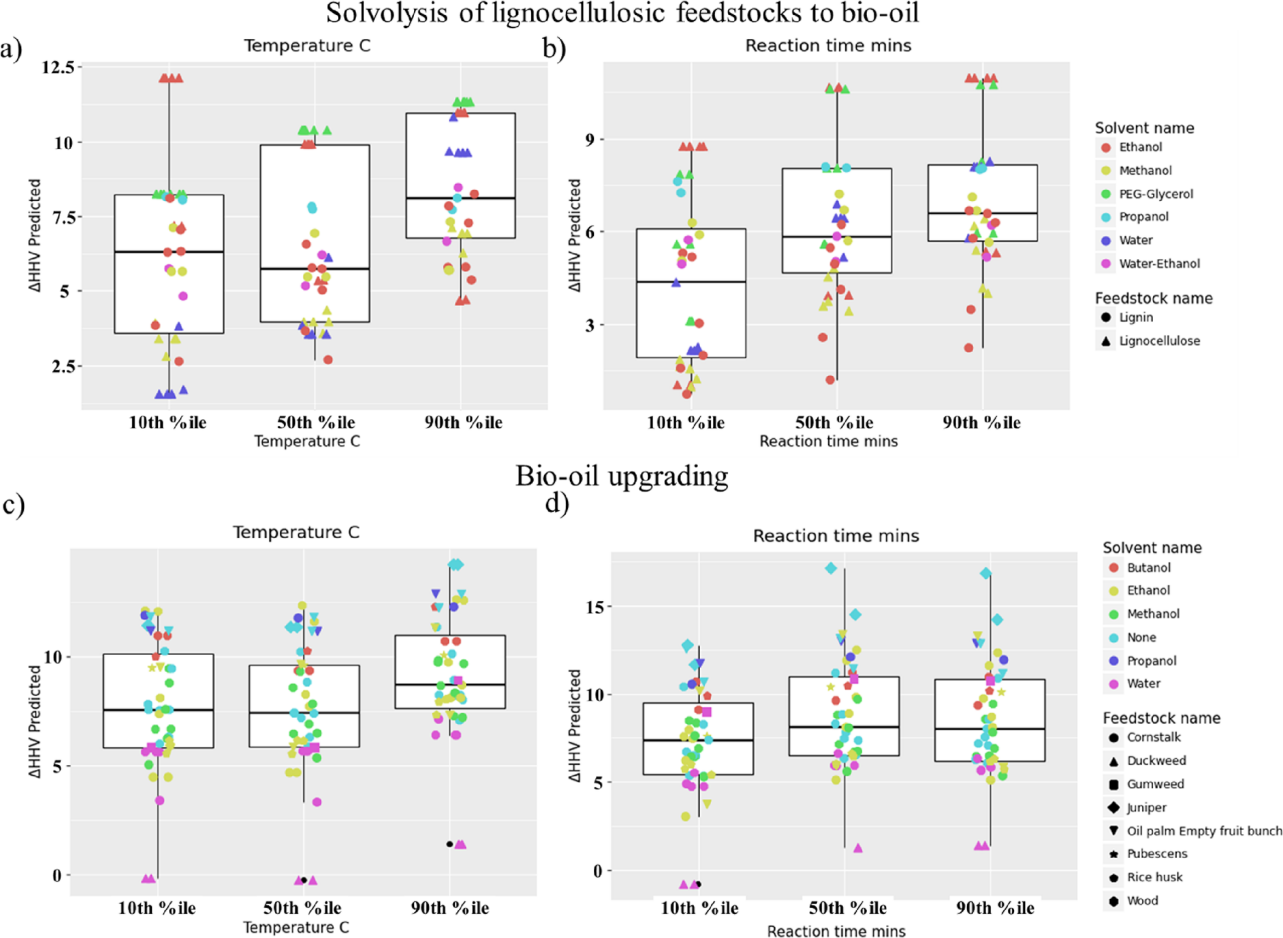
Partial dependency plot for ΔHHV changes at different temperatures and reaction time values for solvolysis of biomass to BO in (a) and (b), with solvents ethanol, methanol, polyethylene (PEG)-glycerol, propanol, water, and water–ethanol mixture, and feedstocks divided into either lignin or lignocellulose. BO upgrading in (c) and (d), with solvents butanol, ethanol, methanol, propanol, water or no solvent, and the following feedstocks: cornstalk, duckweed, gumweed, juniper, oil palm empty fruit bunch, pubescens, rice husk, and wood.

Based on the performance of the model and the distribution of variable importance found, the simulation of hypothetical process conditions can be executed. Among the directly controllable process variables, temperature and reaction time are considered the most impactful ones since both variables are in the top five highest importance in the prediction of ΔHHV in Figure 5. With this in mind, Figure 6 shows partial dependency plots for ΔHHV values using different percentiles of temperature and reaction time, in (a) and (b) for solvolysis of biomass to BO and (c) and (d) for BO upgrading.

In Figure 6a, a significant increase in the resulting ΔHHV of BO produced can be observed when using higher temperatures. Similar to Figure 6b, increasing the reaction time results in a higher expected ΔHHV. However, beyond a certain point, the resulting ΔHHV in the produced BO does not appear to increase further. In the context of the gathered data and the reactions involved, it stands to reason that if HDO reactions are ultimately responsible for the ΔHHV value obtained, all of the removable oxygen-containing species will have reacted at a certain point in the process; thus, no further increase in Δ*H*HV should be possible.

It is also interesting to note that this pattern is largely echoed in Figure 5c,d. In the case of Figure 6c, the increase of ΔHHV appears to be only marginally higher at temperatures in the 90th percentile. Figure 6d also shows a similar pattern as in Figure 6c, which is presumably related to the same HDO reactions previously mentioned.

Because of the heterogeneity of the data set in this research in terms of solvents and catalysts, it must be noted that some of these simulated results may stray significantly from what a real experiment would result in. From the perspective of the solvent used, this is related to the fact that organic solvents may interact differently with the catalyst at a higher temperature, in ways that do not necessarily contribute to the expected change of ΔHHV. Notably, the short-chain alcohols used in the experiments in the data set can undergo aldol condensation at different temperatures,^68^ which can compete with the arguably more favored alkylation reactions^69^ or hydrogen transfer,^70^ which would contribute to higher ΔHHV.

### Significance of Results and Comparison to Other Methodologies for Calculating HHV

The models developed in this research displayed a significant prediction performance in spite of the sparsity of the data set. Because of this, using a data set consisting of experiments only involving the use of a particular solvent or gathering more detailed information regarding the properties of the catalyst used should result in a model with much higher prediction performance. Using SHAP values offers the possibility of interpreting variable importance in a way that could allow us to connect the results with specific reaction mechanisms or phenomena that are responsible for the changes in BO upgrading or solvothermal biomass conversion. This is especially valuable because biomass sources and the BO resulting from their conversion can have a very extensive range of properties. Coupling experimental work with machine learning modeling and its associated explainable variable importance will undoubtedly become powerful tools in the study of biomass conversion in general.

The models shown in this study can estimate final HHV or expected increase in HHV as a function of the process parameters, for the first time, as far as the published literature is concerned, in contrast to the various existing formulas derived from Dulong’s formula for HHV^71,72^ that make use exclusively of the elemental composition of the resulting fuel. Other recent works related to biomass, HHV, and machine learning share parallel with Dulong’s formula in that they use the properties of the biomass or biomass conversion products for calculating HHV,^73,74^ but no process parameters. Those works that do involve process parameters tend to center on arguably simpler processes such as gasification,^75^ pyrolysis,^76^ and hydrothermal treatment,^13^ where the number of potential chemical interactions is lower on account of the absence of catalysts or reactive reaction media, such as various heterogeneous catalysts and organic solvents seen in this study. The models for bio-oil upgrading seen in this study can be used to research the cost–benefit–energy relation between proposed upgrading processes that differ in terms of catalysts, solvents, and starting bio-oil. However, it is ultimately important to note that our model is not based on the entirety of the possible experimental space (all the possible combinations of variables) but rather limited only to the scope of currently available published literature, which may limit its applicability to never-before-tested combinations of reactants or catalyst species. In terms of feedstocks, it must be noted that the models in this study were made with only lignocellulosic biomass and lignin in mind, while other carbon-containing feedstocks (such as municipal waste or sewage sludge) may also be used in solvothermal conversion to BO; this model would most probably generate erroneous predictions for those due to the differences in chemical composition.

### Future Research Direction and Recommendations

Machine learning tools can accelerate the development of biomass conversion processes that normally require extensive work with findings that cannot always be extrapolated to other kinds of biomass. This is due to both the large number of process variables in biomass conversion processes and the wide variability in properties of different biomass feedstocks. There are a number of bottlenecks that must be addressed in order for this to be realized. First, clear guidelines for reporting experimental procedures and minimal suggested characterization of feedstocks and catalysts used should be developed. This is a sentiment shared by other authors with regard to lignin-first biorefining,^77^ though it does not address the usage of machine learning. Second, the experimental work related to thermochemical biomass conversion is, in general, very cumbersome and tends to require high-temperature and pressure-resistant equipment that can be costly. This is a matter that other experimental disciplines of science, such as biology^78^ and organic synthesis,^79^ do not struggle with (as much), where high-throughput experimentation via robotic tools is already available^80^ or upcoming.^81^ A possible solution to this matter could be the development of new experimental methods that require less workup and reactors that can be deployed in large numbers simultaneously. Simple reactors made of high-pressure tubing and caps^82^ are examples of alternatives that could be used in high-throughput experimentation of biomass conversion. However, these reactors may have mass transfer limitations that complicate the extrapolation of the obtained results. Model compounds or mixtures of model compounds could be used to deploy extensive arrays of experiments that can be then modeled and analyzed to obtain insight into what can be expected to happen in a given biomass conversion process.

## Conclusions

The interpretable XGBoost models for the prediction of the HHV of BO from the solvothermal conversion of lignocellulosic biomass and BO upgrading were used. The *R*^2^ scores ranging from 0.77 to 0.86 could be achieved despite the large diversity of reaction conditions, solvents, and types of catalysts found in the data set. SHAP values also provided the interpretable variable importance that coincides with findings found in the literature and highlighted the useful correlations that may allow for useful prediction of the expected BO quality given a set of process variables, minimizing the experimental work needed to obtain meaningful results. This work demonstrates that few variables dictate the possible increase in HHV in a given bio-oil to be upgraded or the conversion of lignocellulosic biomass to bio-oil in terms of its characteristics, such as elemental composition. Statistically speaking, variables such as choice of solvent, initial moisture concentration in bio-oil, and catalyst active phase were shown to be of little importance compared to reaction time and temperature, within the context of this data set.
